# Comparative Transcriptional Analysis of *Lactobacillus plantarum* and Its *ccpA*-Knockout Mutant Under Galactooligosaccharides and Glucose Conditions

**DOI:** 10.3389/fmicb.2019.01584

**Published:** 2019-07-09

**Authors:** Chen Chen, Linlin Wang, Yanqing Lu, Haiyan Yu, Huanxiang Tian

**Affiliations:** Department of Food Science and Technology, Shanghai Institute of Technology, Shanghai, China

**Keywords:** *Lactobacillus plantarum*, galactooligosaccharides, metabolic regulation, transcriptome, catabolite control protein A

## Abstract

Galactooligosaccharides (GOS) are documented prebiotic compounds, but knowledge of the metabolic and regulatory mechanisms of GOS utilization by lactic acid bacteria is still limited. Here we used transcriptome and physiological analyses to investigate the differences in the logarithmic growth phase of *Lactobacillus plantarum* and *L. plantarum ΔccpA* metabolizing GOS or glucose as the sole source of carbohydrate. In total, 489 genes (16%) were differentially transcribed in the wild-type *L. plantarum* grown on glucose and GOS and the value is decreased to 7% due to the loss of *ccpA*. Only 6% genes were differentially expressed when the wild-type and the *ccpA* mutant were compared on GOS. Transcriptome data revealed that the carbon sources significantly affected the expression of several genes, and some of the genes were mediated by CcpA. In particular, *lac* and *gal* gene clusters resembled the corresponding clusters in *L. acidophilus* NCFM that are involved in GOS metabolism, indicating that these clusters may be participating in GOS utilization. Moreover, reverse transcription-PCR analysis showed that GOS-related gene clusters were organized in five independent polycistronic units. In addition, many commonalities were found between fructooligosaccharides and GOS metabolism in *L. plantarum*, including differentially expressed genes involved in oligosaccharide metabolism, conversion of metabolites, and changes in fatty acid biosynthesis. Overall, our findings provide new information on gene transcription and the metabolic mechanism associated with GOS utilization, and confirm that CcpA plays an important role in carbon metabolism regulation in *L. plantarum*.

## Introduction

Prebiotics are non-digestible food ingredients that exert beneficial effect on the host’s health by selectively stimulating the growth and activity of health-promoting bacteria in the gastrointestinal tract (GIT) (Andersen et al., 2012; Arnold et al., 2018). Currently, galactooligosaccharides (GOS) are widely accepted as prebiotics (Macfarlane et al., 2008; Monteagudo-Mera et al., 2016). GOS are oligosaccharides with a degree of polymerization between 2 and 10 galactosyl residues and a terminal glucose moiety (Iqbal et al., 2010; Van Leeuwen et al., 2016; Azcarate-Peril et al., 2017). Due to the nature of the oligomer and the association of β-galactoside linkages, GOS act as prebiotic supplements that particularly promote the growth of lactobacilli and bifidobacteria (Andersen et al., 2011; María et al., 2018).

Although it has long been known that oligosaccharides are a prerequisite for the colonization and probiotic activities of lactic acid bacteria (LAB) in the GIT, the understanding of the molecular basis of GOS uptake and metabolism by the *Lactobacillus* species is quite recent. Using microarray transcriptome analysis, Andersen et al. (2011) identified that the *lac* gene cluster (LBA1457–LBA1469) participates in GOS utilization. Specifically, GOS is transported intact by a galactoside-pentose-hexuronide (GPH)-type LacS permease and hydrolyzed by two cytoplasmic β-galactosidases (LacA of the GH42 family and LacLM of the GH2 family) into glucose and galactose. Glucose and galactose are subsequently metabolized via the glycolytic and Leloir pathways, respectively. Interestingly, although the structures of the *lac* gene cluster differ markedly among LAB (Yong and Klaenhammer, 2015), both *lacS* and *lacA* genes exist in LAB, except in *L. delbrueckii* subsp. *bulgaricus*, indicating that LacS and β-galactosidases of the GH42 family coevolved.

In many bacteria, adaptation to changing carbon sources is achieved through a regulatory mechanism called carbon catabolite repression (CCR). This mechanism ensures that the most profitable carbon sources are used first (Akiyama et al., 2015). The catabolite control protein A (CcpA) is a pleiotropic global regulator of CCR in low-GC gram-positive bacteria (Titgemeyer and Hillen, 2002; Fujita, 2009). When bound to its corepressor, Hpr-Ser-P (Presecan-Siedel et al., 1999; Josef et al., 2006), the complex (CcpA-Hpr-Ser46-P) binds to catabolite-responsive element (*cre*) sites in the promoter regions of various genes to repress or enhance gene expression (Seidl et al., 2009; Zotta et al., 2012; Kremling et al., 2015). Six *cre* sites were found in the cluster related to GOS metabolism in *L. acidophilus* NCFM, suggesting that CcpA is involved in GOS metabolism. However, the exact mechanism is still unknown.

*Lactobacillus plantarum* is a versatile lactic acid bacterium used for the production of several fermented and functional foods (Enan et al., 1996; Capozzi et al., 2012; Goh and Klaenhammer, 2015; Sankar et al., 2018; Zhao et al., 2019). Several strains, moreover, have been recognized and used as probiotics (Zhang et al., 2009; Ricciardi et al., 2015; Pavli et al., 2016; Yadav et al., 2016; Qian et al., 2017; Papadopoulou et al., 2018; Arena et al., 2019; Wang et al., 2019). CcpA plays a key role in carbon metabolism regulation (Muscariello et al., 2001; Lorquet et al., 2004; Goffin et al., 2006; Siezen et al., 2006), growth performances and stress robustness of *L. plantarum* (Muscariello et al., 2011; Mazzeo et al., 2012; Zotta et al., 2012). In particular, the metabolic mechanism of fructooligosaccharides (FOS) in *L. plantarum* has been investigated in our recent studies. These studies showed that CcpA regulates CCR by direct and indirect regulation of FOS-related clusters in *L. plantarum* (Chen et al., 2018; Lu et al., 2018). It has also been reported that different *L. plantarum* strains can effectively utilize GOS (Iqbal et al., 2010; Akiyama et al., 2015); however, the mechanism of GOS metabolism in *L. plantarum* is not yet clear, and the role of CcpA is still unknown. In this study, the glucose and GOS metabolism of *L. plantraum* ST-III and its *ccpA*-knokout mutant (hereafter referred to as *Lactobacillus plantarum ΔccpA*) was investigated through differential transcriptome and physiological analysis. These results will provide insight into the metabolic pathway and regulation of GOS in *L. plantarum* and elucidate the role of CcpA in these processes.

## Materials and Methods

### Organisms, Media, and Growth Conditions

*Lactobacillus plantarum* ST-III was isolated from kimchi and obtained from Bright Dairy & Food Co., Ltd., China. It has many probiotic properties, such as cholesterol removal and strong adhesion to Caco-2 cells (Wang et al., 2011). *L. plantarum ΔccpA* was constructed as described in Chen et al. (2018). Both *L. plantarum* ST-III and *L. plantarum ΔccpA* were cultivated in de Man-Rogosa and Sharpe (MRS) broth (Merck, Darmstadt, Germany) at 37°C in ambient atmosphere under static conditions. For growth experiments, the strains were grown in a chemically defined medium (CDM) (Robert et al., 2000; Teusink et al., 2005) (see Supplementary Table S8) supplemented with filter-sterilized solutions of 1% (w/v) GOS or glucose. The GOS used in the CDM was provided by Quantum Hi-Tech Biological Co., Ltd. (Guangdong, China) and comprised ≥94% GOS (degree of polymerization, DP 2–8, average DP ≈ 3.5), ≤7% lactose, and ≤2% glucose. To exclude the effect of residual sugars on the growth of *L. plantarum*, a mixture of lactose and glucose equivalent to their content in 1% GOS was solely added to the CDM as a control. The results showed that these sugars had no effect on the bacterial growth or the subsequent experiments (data not shown).

### Fermentation and Sampling

For growth experiments, overnight cultures of *L. plantarum* ST-III or *L. plantarum ΔccpA* were transferred with 2% (v/v) inoculum into 500 mL of CDM supplemented with filter-sterilized solutions of 1% (w/v) GOS or glucose. The mixtures were incubated for 16–18 h at 37°C in a bioreactor (Bioflo model 115, New Brunswick Scientific Co., Edison, NJ, United States) and flushed with sterile air (0.1 v/v min), without agitation and controlling the value of pH. During the cells’ growth up to the stationary phase, the samples were withdrawn every 2 h to measure the optical density at 600 nm (OD_600_) for growth analysis. Maximum specific growth rates (μ_max_) were calculated through linear regressions of the plots of ln (OD_600_) versus time during the exponential growth phase (Carvalho et al., 2011). When the OD_600_ reached 0.65 (early logarithmic phase) and 1.5 (early stationary phase), cultures grown on GOS or glucose were harvested by centrifugation (8,000 × *g*, 10 min, 4°C). The cell pellets obtained at OD_600_ of 0.65 were flash frozen for storage at -80°C for further RNA isolation. The supernatants at both sampling points were filtered through a 0.22 μm nylon filter (Titan, China). The sugar consumption and production of organic acids were analyzed by high performance anion exchange chromatography (HPAEC) (Chen et al., 2018) and high-performance liquid chromatography (HPLC), respectively, as previously reported (Lu et al., 2018). Three replicate fermentations were carried out for each treatment.

### RNA Extraction and Transcriptome Analysis

Total RNA was extracted and treated as previously described (Lu et al., 2018). The quality and quantity of RNA were evaluated using Thermo Scientific Nanodrop 2000 and an Agilent 2100 Bioanalyzer, respectively.

Transcriptome sequencing (RNA-seq) was performed on Illumina X10 (Illumina, Inc., San Diego, CA, United States) as previously described (Chen et al., 2018). Briefly, the total RNA was incubated with DNase I at 37°C, rRNA was removed, and cDNA libraries were constructed following a PrimeScript RT reagent kit (Takara, Dalian, China).

### Reverse Transcription Quantitative Polymerase Chain Reaction (RT-qPCR) Verification

Eighteen key differentially expressed genes were subjected to RT-qPCR to validate the RNA-seq data. The amplifications were performed with designed primers (see Supplementary Table S6) using the 7300 Fast Real-Time PCR System (Applied Biosystems). For RT-qPCR analysis, the generated cDNA was mixed with 0.2 mM gene specific primers (see Supplementary Table S6) in a total volume of 25 mL. The PCR cycling conditions were as follows: 95°C for 10 min, followed by 40 cycles of amplification at 95°C for 15 s and 60°C for 30 s. All of the samples were measured in triplicate. The relative gene expression data were analyzed by the 2^-ΔΔCt^ method (Ren et al., 2009) and normalized to the 16S rDNA as the reference gene.

### Target Gene Structure Analysis

Total RNA was extracted and reverse transcribed as described above. Primers were designed based on intergenomic regions spanning two clusters of potentially cotranscriptional genes (*lac* and *gal* gene clusters) (Supplementary Table S7). The chromosomal DNA and total RNA without reverse transcription were used as the templates for positive and negative controls, respectively.

### Bioinformatics Analysis

Reads generated by each sample were mapped to the genome of *L. plantarum* ST-III (accession number: CP002222.1) using Bowtie with a default parameter (Langmead et al., 2009). The raw read count for each gene of the two culture samples was analyzed by DEGseq. The MA-plot-based method with the random sampling model in the DEGseq package was then used to calculate the abundance of the expression of each gene in the two culture samples (Wang et al., 2010). In calculations, DEGseq converts the raw read count to reads per kilo bases per million reads (RPKM). The threshold of the *p*-value for this analysis was determined using the false discovery rate (FDR). “FDR < 0.001 and | normalized fold | > 1” was used as the threshold to judge significance of gene expression differences in the four comparison groups: the wild-type strain grown on GOS versus the wild-type strain grown on glucose, the *ccpA* mutant grown on GOS versus the *ccpA* mutant grown on glucose, the *ccpA* mutant grown on glucose versus the wild-type strain grown on glucose, the *ccpA* mutant grown on GOS versus the wild-type strain grown on GOS.

### Statistical Analysis

Statistical analyses were conducted using SPSS (v. 19.0, IBM SPSS, Chicago, Ill., United States). Duncan’s test was used to determine statistical differences. Differences between samples with a *p*-value of <0.05 were considered to be statistically significant.

## Results

### Growth Profiles of *L. plantarum* and *L. plantarum ΔccpA* on GOS and Glucose

**FIGURE 1:**
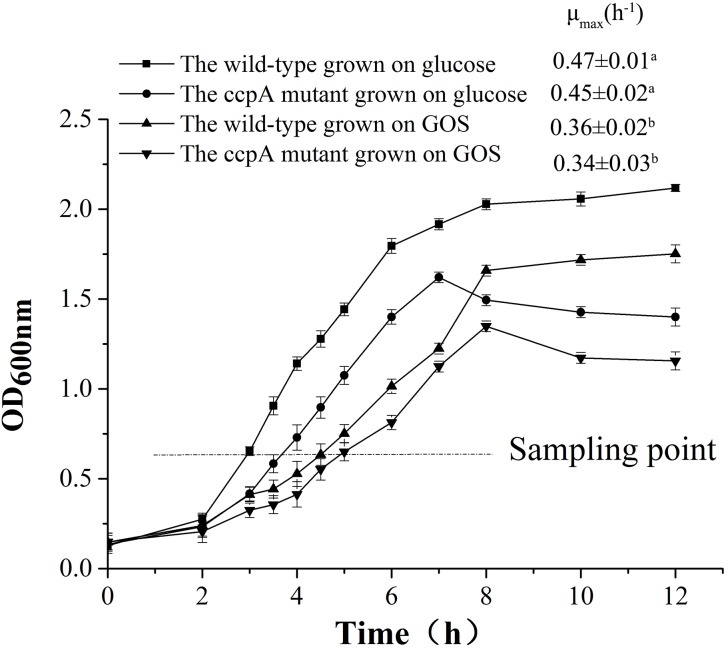
Growth of the wild-type and *ccpA* mutant in a CDM supplemented with 1% commercial GOS and glucose. A sampling point was chosen for the transcriptome analysis, metabolite measurement, and RT-qPCR analysis. The μ_max_ for each condition were also calculated and are shown in the figure. Data presented are mean values based on three replicate fermentations. Error bars indicate standard deviations. Values marked with different lowercase superscript letters (a,b) indicate that they are significantly different (*p* < 0.05) from other values.

*Lactobacillus plantarum* and *L. plantarum ΔccpA* were cultured in CDM containing 1% glucose and GOS at 37°C, and their growth profiles are shown in Figure 1. The maximal specific growth rate (μ_max_) of the GOS-grown wild-type cultures (0.36 ± 0.02 h^-1^) was lower than that of glucose-grown wild-type cultures (0.47 ± 0.01 h^-1^). Compared with the wild-type strain, *L. plantarum ΔccpA* showed a lower growth rate in glucose (μ_max_, 0.45 ± 0.02 h^-1^) and GOS (μ_max_, 0.34 ± 0.02 h^-1^). Notably, the growth tendency of the two strains grown on the same carbon sources showed subtle differences in the stationary phase. The growth of the wild-type strain increased slightly in glucose and GOS, whereas that of its *ccpA* mutant showed a slow declining trend.

### Fermentation Profiles of *L. plantarum* and *L. plantarum ΔccpA* Grown on GOS and Glucose

The levels of sugar consumption and metabolite formation during fermentation with *L. plantarum ST-III* and *L. plantarum ΔccpA* on GOS or glucose are also shown in Table 1. The consumption of GOS is higher than glucose when *L. plantarum* or *L. plantarum ΔccpA* reached the same value of OD_600_, and this phenomenon was more significant at an OD_600_ of 1.5 than at an OD_600_ of 0.65. Lactate and acetate are the main end products of fermentation by *L. plantarum* and *L. plantarum ΔccpA* grown on GOS and glucose. *L. plantarum* grown on GOS produced less lactate and more acetate than that grown on glucose (*p* < 0.05), which is consistent with the finding of *L. plantarum* grown on FOS (Lu et al., 2018). In the absence of CcpA, the levels of acetate and lactate did not vary between the strains grown on GOS or glucose. Notably, the *ccpA* mutant grown on glucose produced more acetate and less lactate than the wild-type strain. This situation also occurred in the presence of GOS, although to a lesser extent. In addition, no difference was observed in the level of formate between the four conditions.

**Table 1:** Comparison of sugar consumption and metabolites resulting from the fermentation of GOS or glucose by wild-type *L. plantarum* ST-III or *L. plantarum ΔccpA* at OD_600_ of 0.65 and OD_600_ of 1.5^1^.

Conditions		Wild-type strain				*ccpA* mutant strain		
		
	Sugar consumption (mM)	Metabolite formation (mM)			Sugar consumption (mM)	Metabolite formation (mM)		
		
		Lactate	Acetate	Formate		Lactate	Acetate	Formate
OD,0.65 Glucose^2,3^	21.72 ± 0.51^Aa^	15.41 ± 0.70^Aa^	3.03 ± 0.21^Aa^	1.32 ± 0.10^Aa^	20.50 ± 0.45^Aa^	10.63 ± 0.06^Ab^	6.72 ± 0.12^Aa^	1.21 ± 0.15^Aa^
GOS	22.43 ± 0.14^Aa^	11.91 ± 0.22^Ba^	6.11 ± 0.05^Ba^	1.20 ± 0.05^Aa^	22.20 ± 0.29^Aa^	10.31 ± 0.16^Aa^	7.23 ± 0.12^Aa^	1.43 ± 0.05^Aa^
OD,1.5 Glucose^2,3^	49.30 ± 0.23^Aa^	35.32 ± 1.32^Aa^	4.72 ± 0.05^Aa^	1.49 ± 0.11^Aa^	46.08 ± 0.37^Aa^	26.50 ± 0.78^Ab^	12.41 ± 0.83^Ab^	1.59 ± 0.09^Aa^
GOS	52.65 ± 0.54^Ba^	28.21 ± 1.12^Aa^	11.43 ± 1.23^Ba^	1.61 ± 0.12^Aa^	51.52 ± 0.46^Ba^	26.28 ± 0.91^Aa^	12.92 ± 0.34^Aa^	1.72 ± 0.21^Aa^


*^1^Data presented are mean values based on three replicate fermentations. Error bars indicate standard deviations. ^*2*^Values marked with different superscript uppercase letters (AB) indicate those are statistically significant differences (*p* < 0.05) between cells grown on GOS and those grown on glucose at an OD_600_ of 0.65 and 1.5. ^*3*^Values marked with different superscript lowercase letters (ab) indicate those are statistically significant differences (*p* < 0.05) between wild-type and *ccpA* mutant at an OD_600_ of 0.65 and 1.5.*

### Transcriptome Profiling of Wild-Type and *ccpA* Mutant Grown on Glucose and GOS

Based on the growth profiles of the strains on GOS and glucose, the early logarithmic phase was selected for transcriptome analysis. Global transcriptional regulation of wild-type and *ccpA* mutant in a CDM of glucose and GOS was analyzed by RNA-seq. The statistical data for the transcriptome analysis is summarized in Supplementary Table S1. For *L. plantarum* ST-III and *L. plantarum ΔccpA* grown on GOS and glucose, the numbers of clean reads obtained were 6.5, 7.0, 6.6, and 5.8 million and the mapping rates were 71.50%, 73.83%, 69.68% and 72.19%, respectively. The average read length was 150 bp (see Supplementary Table S2). In addition, the saturation analysis showed that when the number of reads of both samples reached 1 million, the sequencing was saturated (Tan et al., 2015) (Supplementary Figure S1), and the gene coverage indicated sufficient sequencing depth (Lu et al., 2018) (Supplementary Figure S2).

**FIGURE 2:**
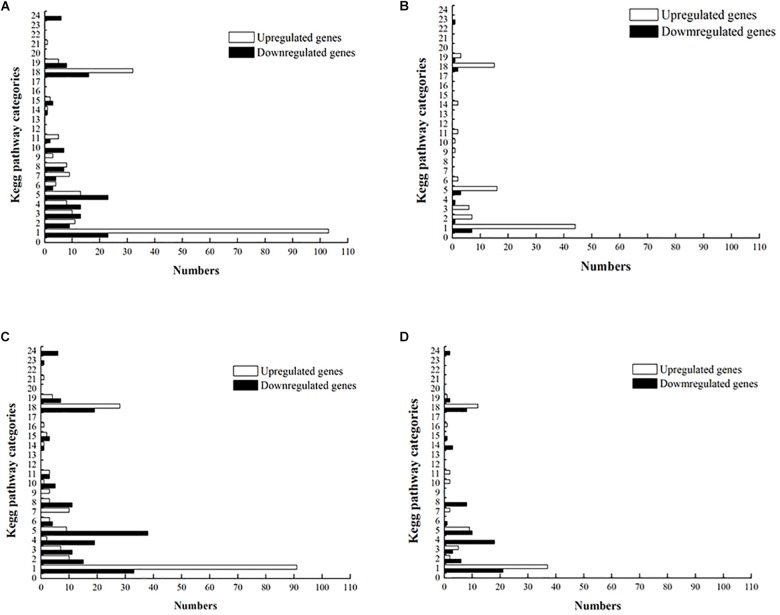
Distribution of upregulated and downregulated genes in the four pair-wise comparisons based on KEGG pathway categories. **(A)** The *ccpA* mutant grown on glucose versus the wild-type strain grown on glucose. **(B)** The *ccpA* mutant grown on GOS versus the wild-type strain grown on GOS. **(C)** The wild-type strain grown on GOS versus the wild-type strain grown on glucose. **(D)** The *ccpA* mutant grown on GOS versus the *ccpA* mutant grown on glucose. KEGG pathway categories: 1, carbohydrate metabolism; 2, energy metabolism; 3, lipid metabolism; 4, nucleotide metabolism; 5, amino acid metabolism; 6, metabolism of other amino acids; 7, glycan biosynthesis and metabolism; 8, metabolism of cofactors and vitamins; 9, metabolism of terpenoids and polyketides; 10, biosynthesis of other secondary metabolites; 11, xenobiotic biodegradation and metabolism; 12, enzyme families; 13, transcription; 14, translation; 15, folding, sorting, and degradation; 16, replication and repair; 17, RNA family; 18, membrane transport; 19, signal transduction; 20, signaling molecules and interaction; 21, transport and catabolism; 22, cell motility; 23, cell growth and death; 24, cellular community – prokaryotes.

Reads extracted from the RNA-Seq data were mapped into the annotated *L. plantarum* ST-III genome (NCBI GI: PRJNA493968), and gene expression was quantified as RPKM sequenced. The mean RPKM of the wild-type and *ccpA* mutant grown on glucose were 419 and 397, respectively, and of wild-type and *ccpA* mutant grown on GOS were 417 and 402, respectively (Supplementary Table S1). To investigate the changes in the gene expression of wild-type and *ccpA* mutant during fermentation, the standard was set to a fold change of >2 and FDR of <0.001 (Zheng et al., 2015) (Supplementary Table S1). The overall transcriptome profiles were compared in four pair-wise comparisons to identify genes that were differentially expressed between wild-type and *ccpA* mutant during GOS or glucose fermentation in CDM. Each differential gene expression profile is described as an XY plot of log_10_RPKM represented by a different color (Supplementary Figure S3). Among the pair-wise comparisons, the transcriptome profiles of the wild-type and *ccpA* mutant on glucose were compared in our previous study (Lu et al., 2018). In the present study, we focused on the following three pair-wise comparisons to analyze GOS metabolism: between wild-type strain grown on glucose and GOS, between *ccpA* mutant grown on glucose and GOS, and between wild-type and *ccpA* mutant grown on GOS. As per the Kyoto Gene and Genomic Encyclopedia (KEGG) classification, differentially expressed genes were divided into multiple functional categories (Figure 2).

In total, 18 key genes were selected for validating the transcriptome analysis data using RT-qPCR (Supplementary Table S3). Although the magnitude of the genetic variation differed between the results of the two analyses, the RT-qPCR results showed similar up- and downregulations to the transcriptome results, confirming the reliability of the transcriptome data.

### Comparison Between the Wild-Type Strain Grown on Glucose and GOS

**FIGURE 3:**
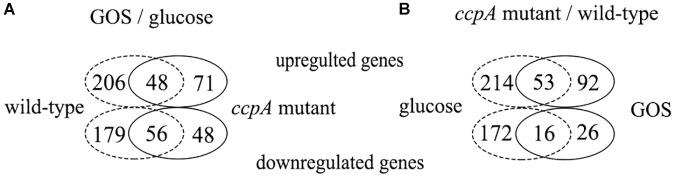
Venn diagrams of the number of genes differentially expressed in the four pair-wise comparisons. **(A)** Number of genes differentially expressed in response to GOS compared with glucose of the wild-type (dotted line) and *ccpA* mutant (full line). **(B)** Number of genes differentially expressed in *ccpA* mutant compared to the wild-type strains, grown on glucose (dotted line) and GOS (full line).

**FIGURE 4:**
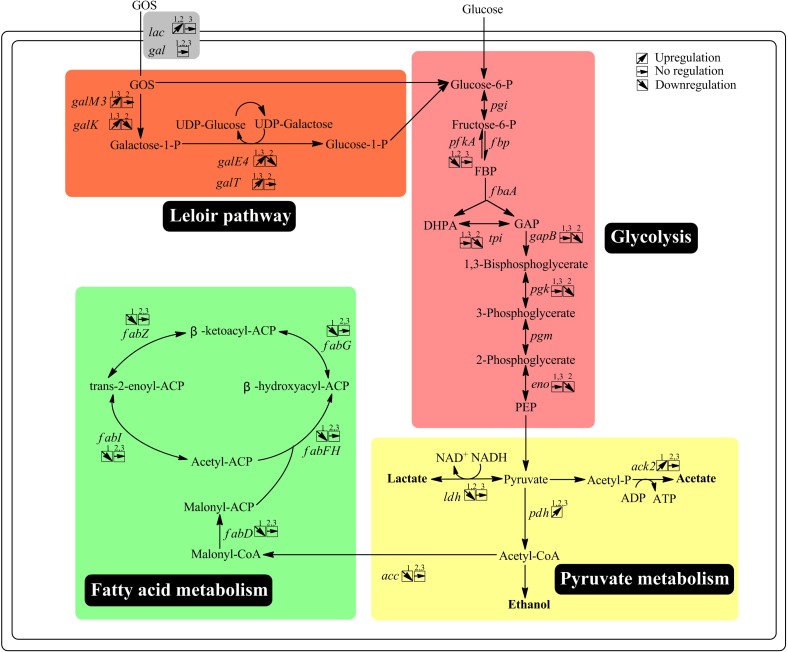
Overview of the key genes with altered expression and their related pathways via transcriptome analysis. 1, The wild-type strain grown on GOS versus the wild-type strain grown on glucose; 2, the *ccpA* mutant grown on GOS versus the *ccpA* mutant grown on glucose; 3, the *ccpA* mutant grown on GOS versus the wild-type strain grown on GOS. Gene annotation downloaded from NCBI: galM3, aldose 1-epimerase; galK, galactokinase; galE4, UDP-glucose 4-epimerase; galT, UDPglucose–hexose-1-phosphate uridylyltransferase; pgi, glucose-6-phosphate isomerase; fbp, fructose-1,6-bisphosphatase; pfkA, 6-phosphofructokinase; fbpA, fructose-bisphosphate aldolase; tpi, triose-3-phosphate isomerase; gapB, glyceraldehyde-3-phosphate dehydrogenase; pgk, phosphoglycerate kinase; pgm, phosphoglycerate mutase; eno, enolase; pyk, pyruvate kinase; ldh, L-lactate dehydrogenase; pfl, pyruvate formate-lyase; pdh, pyruvate dehydrogenase; pox, pyruvate oxidase; pta, phosphotransacetylase; ack, acetate kinase; acc, acetyl-CoA carboxylase; fabD, ACP-S-malonyltransferase; fabF, 3-oxoacyl-ACP synthase II; fabH, 3-oxoacyl-ACP synthase III; fabG, 3-oxoacyl-ACP reductase; fabZ, (3R)-hydroxymyristoyl-ACP dehydratase; fabI, enoyl-ACP reductase.

Supplementary Table S1 lists the genes differentially expressed between wild-type strain grown on glucose and GOS. In total, 489 genes (16% of all genes) were found to be differentially expressed, 254 of which were upregulated and 235 were downregulated (Figure 3A). In the three pair-wise comparisons of this study, this result indicates the largest number of genetic changes. Among the differentially expressed genes, 181 genes were classified into specific expression pathways based on the KEGG database. Notably, most of the differentially expressed genes (35%) were involved in carbohydrate metabolism, including the phosphotransferase system (PTS), galactose metabolism, pyruvate metabolism, fatty acid biosynthesis, and other such processes (Figure 4).

Notably, 30 PTS genes were differentially expressed in this comparison, reflecting potential changes in the uptake of glucose, galactose, fructose, and cellobiose. In comparison with the wild-type strain grown on glucose, one operon of mannose-specific transporter (LPST_C0484, LPST_C0486) was downregulated (2.03–2.26-fold) and the following six genes were upregulated (2.03–32.67-fold) by GOS: mannitol-specific transporter (LPST_C0187, LPST_C0189), cellobiose-specific transporter (LPST_C0363, LPST_C0366, LPST_C2476–LPST_C2477), ascorbate-specific transporter (LPST_C2890-LPST_C2892), galactosamine-specific transporter (LPST_C2175–LPST_C2177), galactitol-specific transporter (LPST_C2897–LPST_C2898), and glucitol/sorbitol-specific transporter (LPST_C2982–LPST_C2983) (Supplementary Table S4). These findings suggest that GOS and its derivatives modify the expression of genes involved in the PTS.

The expression of genes related to pyruvate metabolism and fatty acid metabolism was also altered by GOS metabolism. The genes encoding pyruvate dehydrogenase (LPST_C1775- LPST_C1778), pyruvate formate lyase (LPST_C2728) and pyruvate oxidases (LPST_C2161, LPST_C2933) were found to be upregulated in the presence of GOS in this study, while the genes encoding acetyl-CoA carboxylase (LPST_C1533, LPST_C1535-LPST_C1537), L-lactate dehydrogenase (LPST_C0295), and pyruvate kinase (LPST_C1523) were significantly downregulated. Differential expression of these genes was associated with pathways in which lactate is converted to acetate and formate, consistent with our previous findings (Chen et al., 2015). Furthermore, in accordance with our previous study, the expression of 10 genes (LPST_C1327–LPST_C1328, LPST_C1330–LPST_C1338) involved in fatty acid biosynthesis showed at least 3.87-fold downregulation in the presence of GOS compared with their expression in the presence of glucose (Supplementary Table S5) (Chen et al., 2015).

**FIGURE 5:**
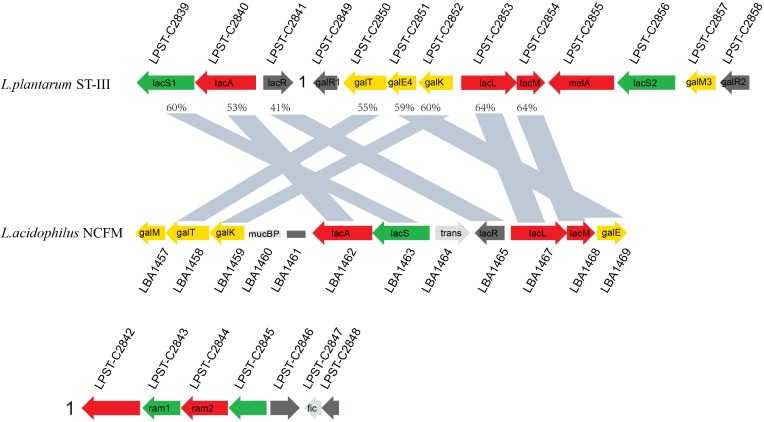
Structural alignment of gene clusters identified within genome-sequenced lactobacilli and related LAB. Putative functions are indicated by color: carbohydrate permeases (green), transcriptional regulators (black), glycoside hydrolases (red), Leloir pathway (yellow), and genes not related to carbohydrate metabolism (gray). The similarity of the corresponding gene is marked on the graph.

As indicated by the transcriptome data, GOS induced significant upregulation (71.51–148.06-fold) in the expression of a cluster (LPST_C2839–LPST_C2841; designated as the *lac* cluster) comprising genes encoding a GPH permease (LacS1), a β-galactosidase (LacA), and a transcriptional regulator (LacR1). In addition, the expression of another related gene cluster (LPST_C2849–LPST_C2858; designated as the *gal* cluster) increased by 2.93–284.05-fold (Supplementary Table S4). The organization of these two clusters (*lac* and *gal*) was compared between *L. plantarum* ST-III and *L. acidophilus* NCFM by BLASTp analysis to investigate the similarity between their structures (Figure 5). The identified GPH permease LacS1 (LPST-C2839) of *L. plantarum* ST-III showed 60% protein sequence identity with the lactose permease of *L. acidophilus* NCFM. Similarly, the two β-galactosidases LacA (LPST-C2840) and LacLM (LPST-C2853–LPST-C2854) of *L. plantarum* ST-III showed 53% and 64% protein sequence identity with their counterparts in *L. acidophilus* NCFM, respectively. The genes involved in Leloir pathway, including *galT* (LPST-C2850), *galE4* (LPST-C2851) and *galK* (LPST-C2852), exhibited 55%, 59% and 60% similarity between the two strains, respectively. However, two components, *melA* (LPST-C2855) and *lacS2* (LPST-C2856), which encodes an alpha-galactosidase and a major facilitator superfamily transporter, appeared only in *L. plantarum*. These results indicate that the clusters *in L. plantarum* and *L. acidophilus* NCFM have similar structures with a high degree of homology, suggesting that the *lac* and *gal* clusters are involved in GOS transport and utilization in *L. plantarum*.

**FIGURE 6:**
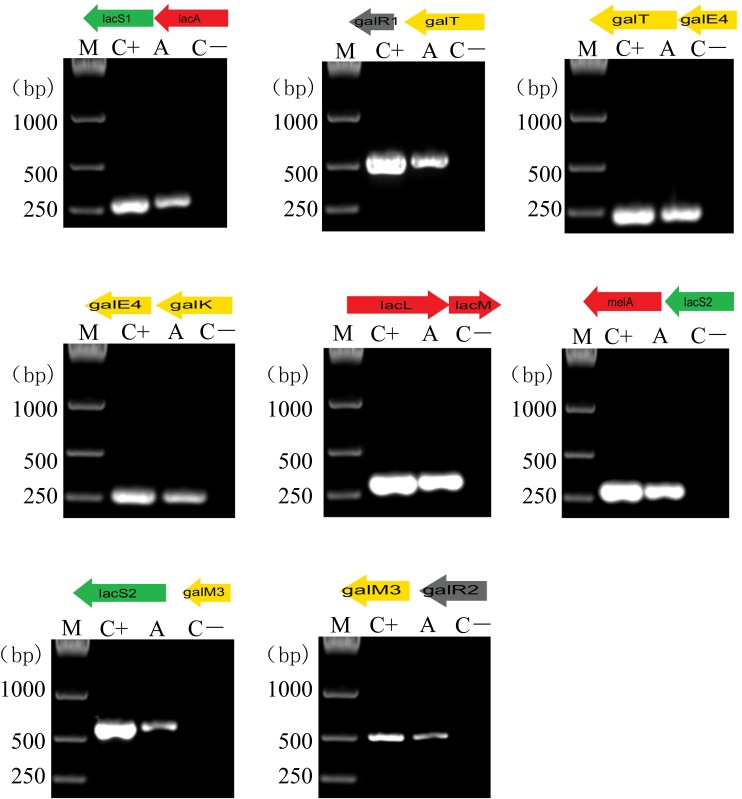
RT-PCR analysis of the transcriptional organization of *lac* and *gal* clusters in *L. plantarum* ST-III. M, DNA size marker; C+, positive control using chromosomal DNA of the strain as the template; A, fragment amplified with a specific primer set using cDNA generated from GOS-induced *L. plantarum ST-III* as the template; C-, negative control using total RNA without the reverse transcription step. Primers used are listed in Supplementary Table S7.

Furthermore, successive overlapping RT-PCR amplifications of polycistronic operon structures were performed for the analysis of the cotranscription of 13 genes (Figure 6). As previously described, cDNA obtained by reverse transcription of the total RNA extracted from GOS-induced culture was used as the PCR template (Chen et al., 2018). The results demonstrated that the *gal* cluster was transcribed into three polycistronic units: *galR1, galT, galE4*, and *galK* transcribed together; *melA, lacS2, galM3*, and *galR2* cotranscribed as an operon; and *lacL* and *lacM* divergently oriented and transcribed together. As expected, the *lac* cluster was transcribed into two transcriptional units: *lacS1* and *lacA* cotranscribed as an operon and *lacR* transcribed alone (Figure 6).

### Comparison Between the *ccpA* Mutant Grown on GOS and Glucose

Next, the transcriptome of the *ccpA* mutant in response to glucose and GOS was compared. Compared with the *ccpA* mutant grown on glucose, 223 genes (7% of all genes) were found to be differentially expressed in the *ccpA* mutant grown on GOS, 119 of which were upregulated and 104 were downregulated. Notably, 89 of the 223 genes could be clustered into special pathways (Figure 3A). Compared with the gene expression in the comparison between the wild-type strain grown on GOS and glucose, the significantly regulated genes in this comparison was significantly reduced, indicating that some differentially expressed genes were mainly regulated by CcpA. For instance, expression of almost none of the genes associated with pyruvate metabolism and fatty acid synthesis differed in this comparison as only functional CcpA can mediate their differential expression. Nonetheless, most of the differentially expressed genes (65%) were also involved in carbohydrate metabolism (Figure 4). The differentially expressed genes related to carbohydrate metabolism, especially the PTS (only 10 genes), also significantly decreased under this comparison; these PTS were mainly involved in the transport of beta-glucose and mannitol (Supplementary Table S6). For glycolysis, most of the genes in this comparison showed the same changes relative to that of the wild-type grown on glucose and GOS.

As observed from the previous comparison, two gene clusters (*lac* and *gal*) were upregulated in the wild-type strain grown on GOS compared with that grown on glucose. After CcpA knockout, GOS increased the expression of the *lac* gene cluster by at least 60.93-fold as well as that of the *gal* cluster, albeit to a lesser extent (at least 9.33-fold) (Supplementary Table S5). These results indicate that these genes may be controlled by regulators other than CcpA.

### Comparison Between the Wild-Type and *ccpA* Mutant Grown on GOS

As reported previously, CcpA plays a key role in carbohydrate metabolism regulation in *L. plantarum* and functions in a glucose-dependent manner (Chen et al., 2018); thus, the difference in the transcriptome profile between wild-type and *ccpA* mutant was low (6%) could be expected. Further, compared with the wild-type strain, approximately 187 genes (6%) were differentially expressed in the *ccpA* mutant at least by 2-fold in the presence of GOS (*p* < 0.05), 145 of which were upregulated and 42 were downregulated (Figure 3B). Among these 187 genes, only 81 could be mapped to the metabolic pathways. Most of these genes were also mapped to carbohydrate metabolism (37%), similar to those in the wild-type and *ccpA* mutant grown on glucose (Chen et al., 2015). These genes are mainly involved in the PTS, pyruvate metabolism, glycolysis, and fructose and mannose metabolism (Figure 4).

Due to the loss of CcpA, GOS could also induce the expression of some genes encoding PTS components (2.35–20.20-fold increase), including sucrose-specific transporter, cellobiose-specific transporter, and galactitol-specific transporter. This manifested that these genes were also inhibited by GOS in the absence of CcpA. Notably, the expression of the *lac* and *gal* clusters did not differ significantly between the wild-type and *ccpA* mutant under the GOS condition. As both strains were grown on GOS, the genes involved in GOS metabolism were expressed similarly in both strains.

Similar to that observed in the comparison of wild-type and *ccpA* mutant under the glucose condition, some genes involved in the metabolism of pyruvate, including components of pyruvate dehydrogenase (LPST_C1776- LPST_C1778) and pyruvate oxidases (LPST_C2161), displayed similar trends in the presence of GOS. Interestingly, the gene (LPST_C0172) encoding acetate kinase was downregulated in the glucose condition but significantly upregulated in the GOS condition with CcpA inactivation. These results suggested that the presence of GOS could switch the fermentation pattern from homofermentation to heterofermentation, although to a lesser extent. The results of metabolite production confirm this hypothesis.

## Discussion

The prebiotic effect of GOS on the enteric populations has been confirmed *in vivo* and *in vitro* (Burr et al., 2010; Andersen et al., 2011; Mazzeo et al., 2012; Akiyama et al., 2015; Safari and Paolucci, 2017). Moreover, the role of CcpA as a global regulator has been revealed in many carbon metabolism regulatory mechanisms in gram-positive bacteria (Zotta et al., 2012; Chen et al., 2018; Lu et al., 2018). However, our knowledge of the mechanism of GOS metabolism in *L. plantarum*, especially of the regulatory mechanism of CcpA, is still limited. In the present study, the whole transcriptome, growth profiles, and metabolite production of *L. plantarum* ST-III and *L. plantarum ΔccpA* were analyzed using GOS or glucose as the sole carbohydrate source to reveal the global changes in response to carbon sources and availability.

The presence of GOS significantly affected the growth of *L. plantarum* ST-III, consistent with the findings of its growth on FOS in our previous study (Chen et al., 2015). The μ_max_ results indicated that the wild-type strain grown on oligosaccharides, including FOS and GOS, showed significantly reduced growth compared with that grown on glucose. After *ccpA* deletion, μ_max_ further declined in the *ccpA* mutant compared with that in the wild-type strain grown on the same carbon sources. Notably, the absence of *ccpA* (mutant strain) leaded to a slow downward growth trend in the stationary phase in both carbon sources, whereas the presence of *ccpA* (wild-type strain) slowly increased the growth. These results of growth experiment indicate that the growth of *L. plantarum* differed between GOS and glucose and that CcpA was a key contributor to this difference. Whole transcriptome sequencing better revealed the differential gene expression of *L. plantarum* ST-III grown on different carbon sources. Among the four pair-wise comparisons, the maximum number of differentially expressed genes was observed in the comparison of the wild-type strain grown on glucose and GOS, whereas the comparison between the *ccpA* mutant grown on glucose and GOS showed fewer differentially expressed genes. These results confirm that CcpA plays a key role in carbohydrate metabolism regulation in *L. plantarum*. Furthermore, the lowest number of differentially expressed genes was observed in the comparison of wild-type and *ccpA* mutant under the GOS condition. Studies have shown that carbohydrate metabolism regulation in *L. plantarum* is mainly mediated in a glucose-dependent manner (Gorke and Stulke, 2008; Cai et al., 2012). Although GOS is considered to be a poorly metabolized carbohydrate, it can also trigger the regulation of carbon catabolic metabolites (Zeng et al., 2013).

**FIGURE 7:**
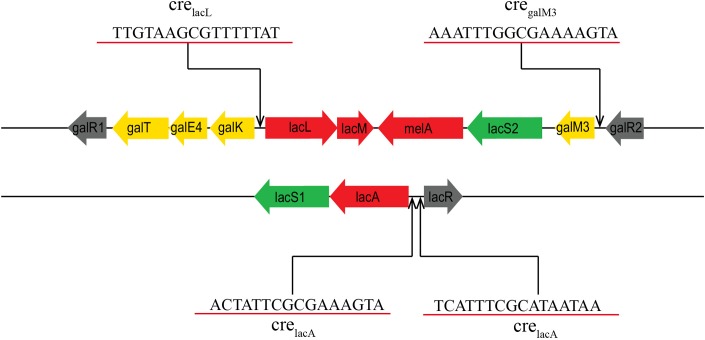
Prediction of the potential *cre* sites in the *gal* and *lac* clusters of *L. plantarum* ST-III. Putative functions are indicated by color: carbohydrate permease (green), transcriptional regulators (black), glycoside hydrolases (red), Leloir pathway (yellow). Putative *cre* sites are underlined in red.

As mentioned above, almost all genes of the *lac* and *gal* clusters were significantly upregulated (2.93–284.05-fold) in *L. plantarum* ST-III using GOS or glucose as the sole carbohydrate source. This finding indicates that these two clusters may be involved in GOS metabolism. Furthermore, BLAST analysis revealed that these clusters are similar to those of *L. acidophilus* NCFM, with the exception that *L. plantarum* has two more copy of the *melA* gene and *lacS2* gene. RT-PCR analysis of the transcriptional organization of these gene clusters indicated that the gene cluster associated with GOS metabolism comprises five independent polycistronic units. Previous studies have shown that in the presence of a preferred carbon source, usually glucose, CcpA can effectively bind to *cre* sites to inhibit or activate the transcription of non-preferred metabolism genes (Huichun et al., 2012; Wu et al., 2015). In this study, four *cre* sites were identified among the two clusters (Figure 7), suggesting that the upregulation of these clusters is attributable to the binding of CcpA to *cre* sites within or downstream of the promoter. Notably, the two clusters were also upregulated (9.33–60.93-fold) when comparing *ccpA* mutant cells grown on GOS and glucose, indicating that except for CcpA, GOS metabolism may also be affected by other local regulators. Furthermore, three genes encoding the regulatory factors of LacI family, including *lacR1* (LPST-C2841), *galR1* (LPST-C2849), and *galR2* (LPST-C2858), were found in the gene clusters associated with GOS metabolism. A detailed investigation of the GOS regulatory mechanism in *L. plantarum* is currently underway.

One interesting outcome of this study concerns pyruvate metabolism. Genes related to the proposed pathways for the production of acetate and formate were upregulated in the presence of GOS versus glucose. The measurement of metabolites showed that cells grown on GOS altered their metabolism to produce more acetate and less lactate. These results were similar to our previous study with *L. plantarum* grown on FOS (Chen et al., 2015). Furthermore, after CcpA knockout, the *ccpA* mutant grown on glucose or GOS produced more acetate and less lactate than the wild-type strain. These results confirm that the inactivation of *ccpA* leads to a shift from homolactic fermentation to mixed fermentation. It is suggested that the shift from homolactic fermentation to mixed fermentation by *L. plantarum* is affected by many factors, e.g., *ccpA* inactivation, sugar exhaustion and aerobic condition (Lorquet et al., 2004; Mazzeo et al., 2012; Zotta et al., 2012). As these factors all existed in the present work, thus it can be concluded that the metabolic shifts observed could be attributed to the interaction of these factors.

The transcriptome data suggested that there are several common characteristics between FOS and GOS metabolisms in *L. plantarum*. For example, the gene clusters involved in oligosaccharide metabolism were significantly upregulated in the presence of FOS or GOS, suggesting that these genes are induced for oligosaccharide utilization. In addition, the transcriptome profiles indicated that the fatty acid synthesis-related genes were significantly downregulated in the wild-type cells grown on GOS compared with those on glucose. The result is consistent with the finding of our previous study that oligosaccharide metabolism decreases FA synthesis (Chen et al., 2015). Interestingly, this phenomenon was eliminated after the *ccpA* gene was knocked out, which suggested that CcpA also has an important effect on the regulation of fatty acid metabolism (Lu et al., 2018). Furthermore, the differentially expressed genes of pyruvate metabolism and measurement of metabolites in *L. plantarum* grown on GOS were also similar to our previous studies using FOS as the carbon source (Chen et al., 2015; Lu et al., 2018). In summary, many similar phenomena and changes were observed in the metabolism of different oligosaccharides in *L. plantarum*, including differential expression of related metabolic genes, conversion of organic acids, and changes in fatty acid metabolism, and CcpA was found to play an important role in these processes. Thus, our results suggest that *L. plantarum* exhibits a similar adaptation mechanism in response to oligosaccharides, but the detailed mechanism needs to be investigated in future studies.

## Conclusion

In conclusion, we performed transcriptome and metabolite analyses of *L. plantarum* and *L. plantarum ΔccpA* grown on glucose and GOS. The results demonstrated that the *lac* and *gal* gene clusters resemble the corresponding clusters in *L. acidophilus* NCFM that are involved in GOS utilization and that the same clusters may participate in GOS metabolism in *L. plantarum*. In addition, these clusters contain five independent polycistronic units and four potential *cre* sites for CcpA binding. Meanwhile, we found many common characteristics between FOS and GOS metabolisms in *L. plantarum*, including the induction of oligosaccharide-related genes, conversion to mixed fermentation, and changes in fatty acid biosynthesis. The data obtained in this *in vitro* study provide insight about the mechanism of GOS metabolism in LAB and contribute to the elucidation of complex regulatory networks that function in response to different carbon sources and availability.

## Data Availability

Publicly available datasets were analyzed in this study. This data can be found here: https://www.ncbi.nlm.nih.gov/search/all/?term=+PRJNA493968.

## Author Contributions

CC wrote the manuscript and performed the statistical analyses. LW analyzed the growth and transcriptome of wild-type and *ccpA* mutant in response to glucose and GOS. YL constructed the *ccpA* mutant and analyzed the generalizability of oligosaccharide metabolism. HY performed the RT-PCR analysis, BLASTp analysis, and putative *cre* site prediction. HT designed the research.

## Conflict of Interest Statement

The authors declare that the research was conducted in the absence of any commercial or financial relationships that could be construed as a potential conflict of interest.
