# Supplementary material for: Comparative Transcriptional Analysis of Lactobacillus plantarum and Its ccpA-Knockout Mutant Under Galactooligosaccharides and Glucose Conditions
Source: Front Microbiol. 2019 Jul 9;10:1584. doi: 10.3389/fmicb.2019.01584 (PMC6629832; doi:10.3389/fmicb.2019.01584)
Supplement: Supplementary file 2 [file Data_Sheet_1.PDF]

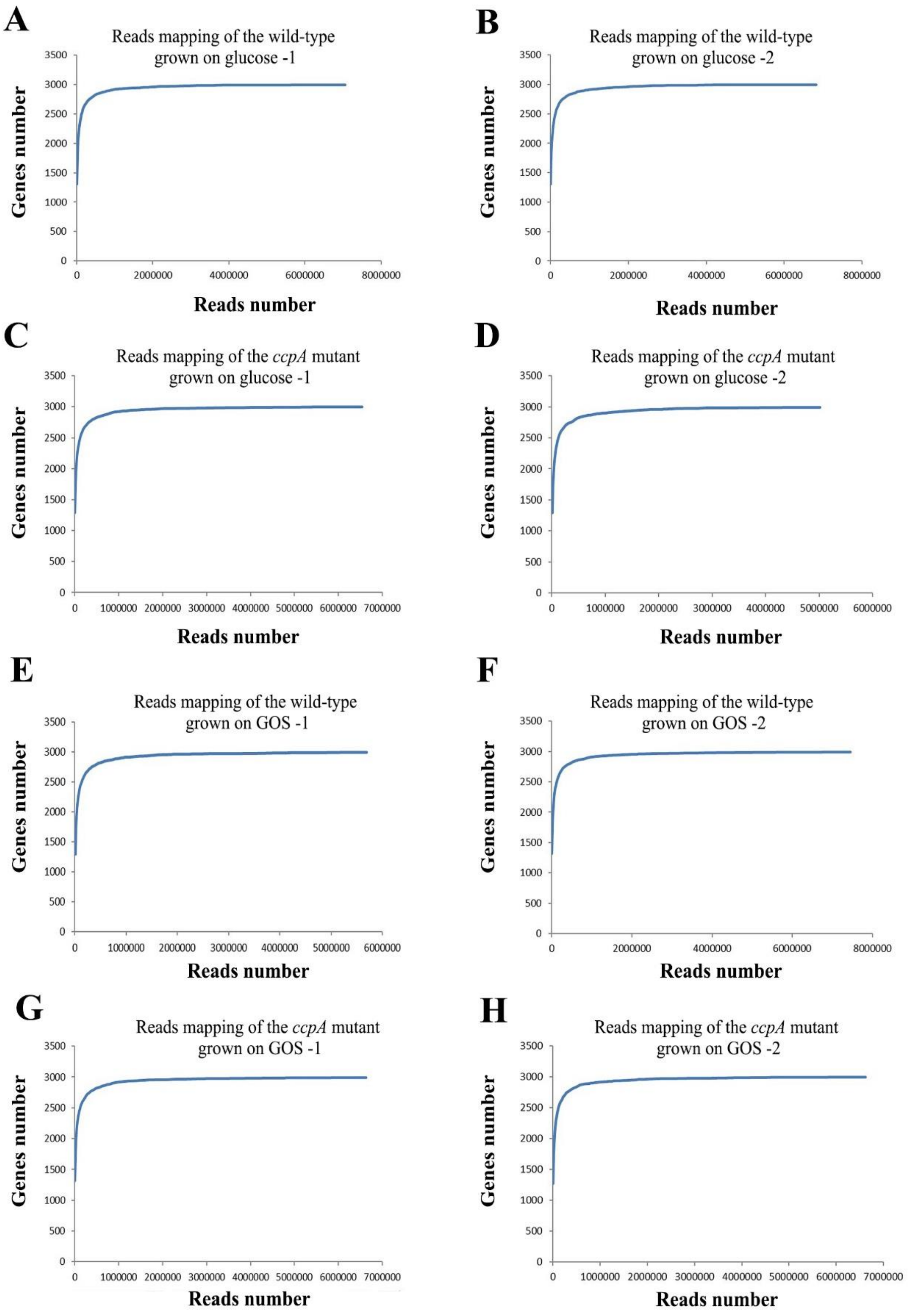

Supplementary Figure 1. The sequencing saturation analysis of transcriptome data in the four conditions. Two replicate fermentations were performed for each treatment, and eight saturation curves were generated.
