# Supplementary material for: Comparative Transcriptional Analysis of Lactobacillus plantarum and Its ccpA-Knockout Mutant Under Galactooligosaccharides and Glucose Conditions
Source: Front Microbiol. 2019 Jul 9;10:1584. doi: 10.3389/fmicb.2019.01584 (PMC6629832; doi:10.3389/fmicb.2019.01584)
Supplement: Supplementary file 3 [file Data_Sheet_2.PDF]

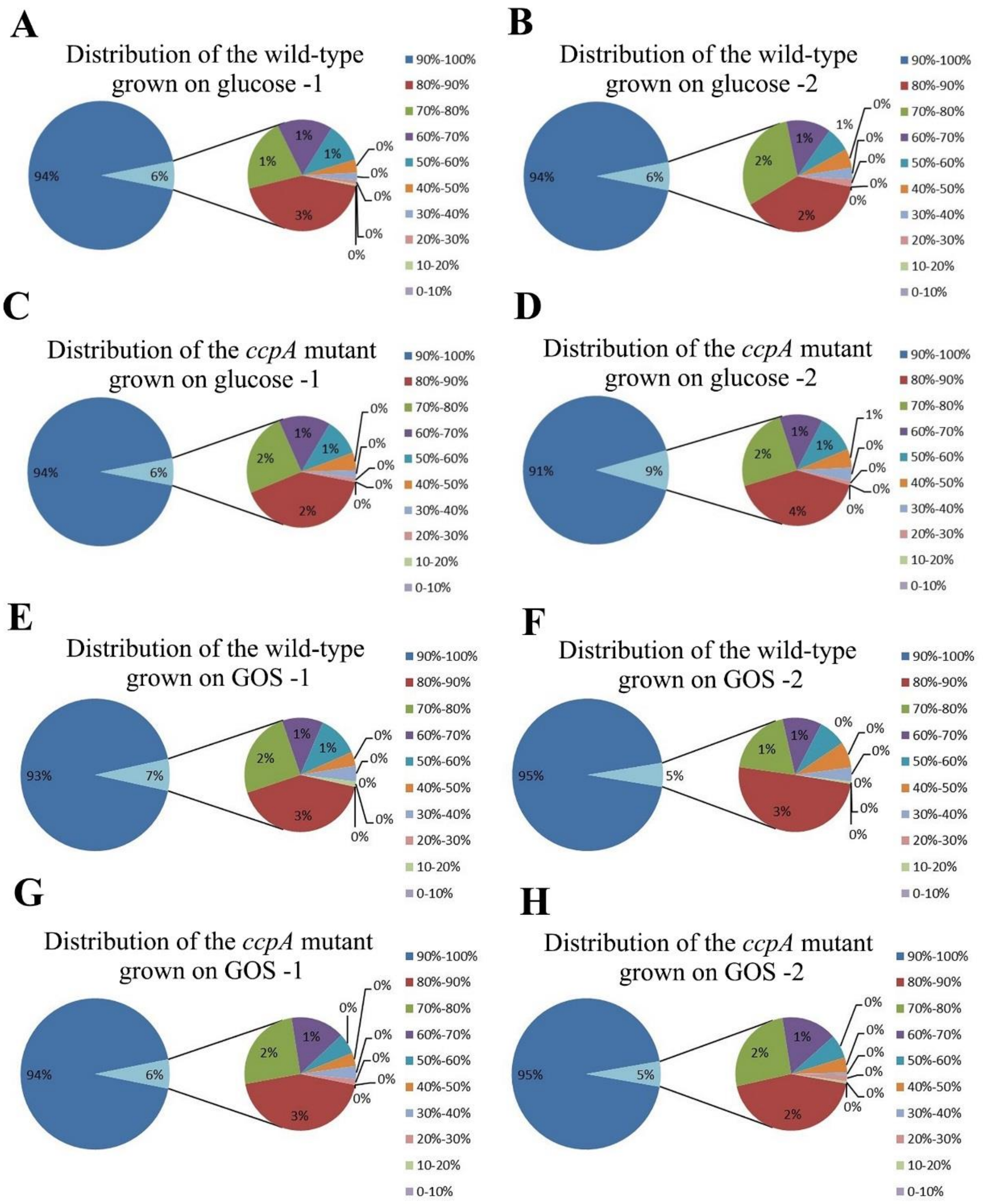

Supplementary Figure 2. Gene coverage distribution of the transcriptome data in the four conditions. The percentage indicates the ratio of the number of genes in different coverage intervals to the total number of genes.
