# Supplementary figures and images for: Comparative Transcriptional Analysis of Lactobacillus plantarum and Its ccpA-Knockout Mutant Under Galactooligosaccharides and Glucose Conditions
Source: Front Microbiol. 2019 Jul 9;10:1584. doi: 10.3389/fmicb.2019.01584 (PMC6629832; doi:10.3389/fmicb.2019.01584)

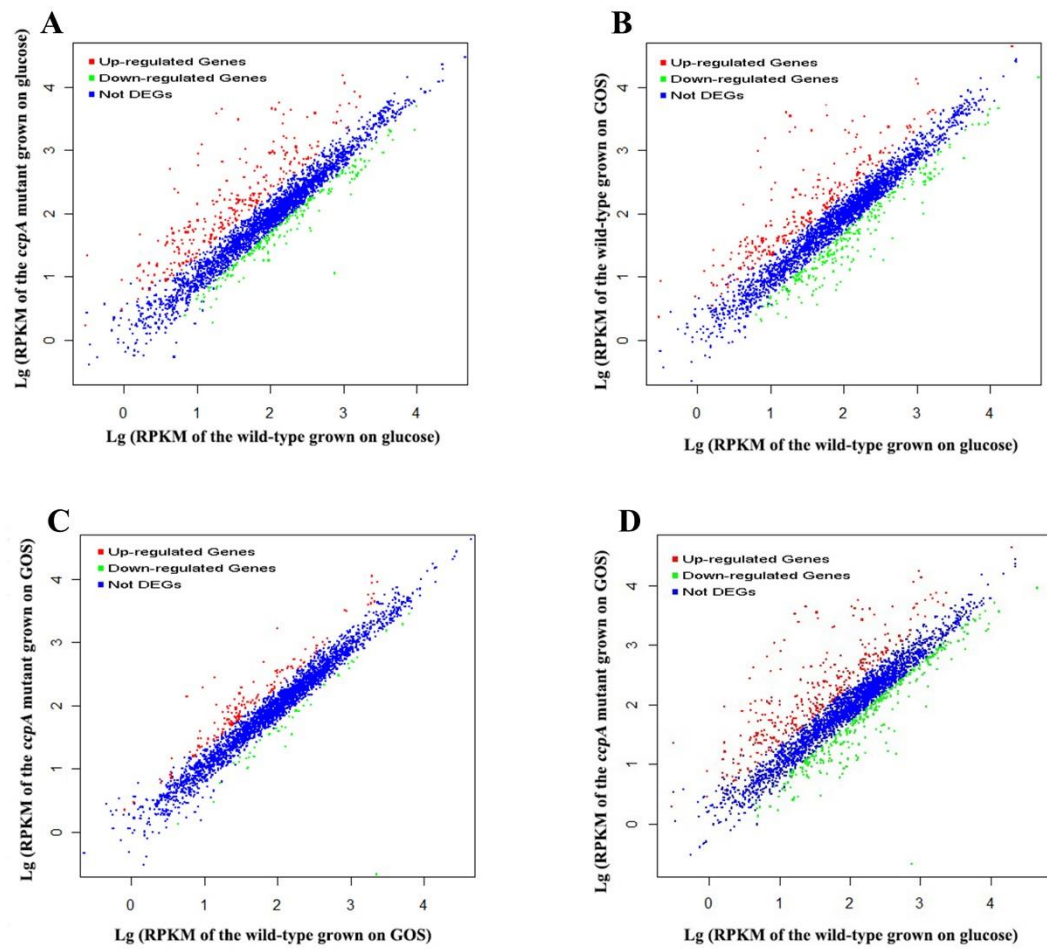

**Supplementary Figure 3.** Representative volcano plots of four pair-wise comparisons.

Supplement: Supplementary file 4 [file Data_Sheet_3.PDF]
